# Associative Self-Anchoring Interacts with Obtainability of Chosen Objects

**DOI:** 10.3389/fpsyg.2015.02012

**Published:** 2016-01-11

**Authors:** Charlotte Prévost, Niall Bolger, Dean Mobbs

**Affiliations:** Department of Psychology, Columbia University, New YorkNY, USA

**Keywords:** self-esteem, associative self-anchoring, psychological reactance, decision-making

## Abstract

While there is evidence that implicit self-esteem transfers to chosen objects (associative self-anchoring), it is still unknown whether this phenomenon extends to explicit self-esteem. Moreover, whether the knowledge that these objects might belong to the self in the future or not affects the evaluation of these objects has received little attention. Here, we demonstrate that evaluations of chosen objects are further enhanced when they are obtainable as compared to when they are not in participants with high explicit self-esteem, whereas participants with low explicit self-esteem exhibit the opposite pattern. These findings extend previous results and shed new light on the role of self-esteem in altering preferences for chosen objects depending on their obtainability.

## Introduction

Self-esteem promotes ‘positive illusions’ such as overly positive self-evaluations, exaggerated perceptions of control and unrealistic optimism; resulting in increased well-being and mental health (Taylor and Brown, 1988). Consistent with the idea that people with a higher sense of self-esteem are better at deceiving themselves, self-esteem has also been suggested to play a key role in the enhancement of valuation of chosen objects. For instance, it has been shown that when choosing an object, an association between the self and the object is formed, a phenomenon referred to as associative self-anchoring (Cadinu and Rothbart, 1996; Gawronski et al., 2007). Consequently, evaluations of the self transfer to the chosen object such that the evaluation of the chosen object is modulated by implicit self-esteem rather than cognitive dissonance (Gawronski and Strack, 2004; Gawronski et al., 2007). For instance, Gawronski et al. (2007) asked participants to make implicit evaluations of two pictures before choosing between them, and showed that implicit evaluations of the chosen picture depend on implicit evaluations of the self. Implicit self-esteem is commonly measured using an initials preference task where participants are asked to rate the likeability of all letters of the alphabet presented in a random order on a 1–5 Likert scale. The preference for one’s own initials is subsequently interpreted as an index of implicit self-esteem. Conversely, explicit self-esteem is measured using questionnaires asking participants to directly reflect on their self-evaluation (e.g., by indicating on a 1–7 Likert scale how much they agree with the following statement: “I have high self-esteem"). Thus, while implicit self-esteem refers to an automatic or unconscious self-evaluation, explicit self-esteem refers to a more conscious and reflective evaluation. Yet, it is still unknown whether associative self-anchoring extends to explicit self-esteem.

Low self-esteem has been associated with an increased tendency to exhibit reactant behavior in the case of freedom elimination (Joubert, 1990). According to psychological reactance theory, eliminating a behavioral freedom previously established will induce a motivational state aimed at restoring the lost freedom, such that a chosen object no longer obtainable will be perceived as more attractive (Brehm, 1966; Brehm and Brehm, 1981). However, whether self-esteem differentially affects the evaluation of chosen objects based on the opportunity of actually receiving these objects has remained untested.

**FIGURE 1:**
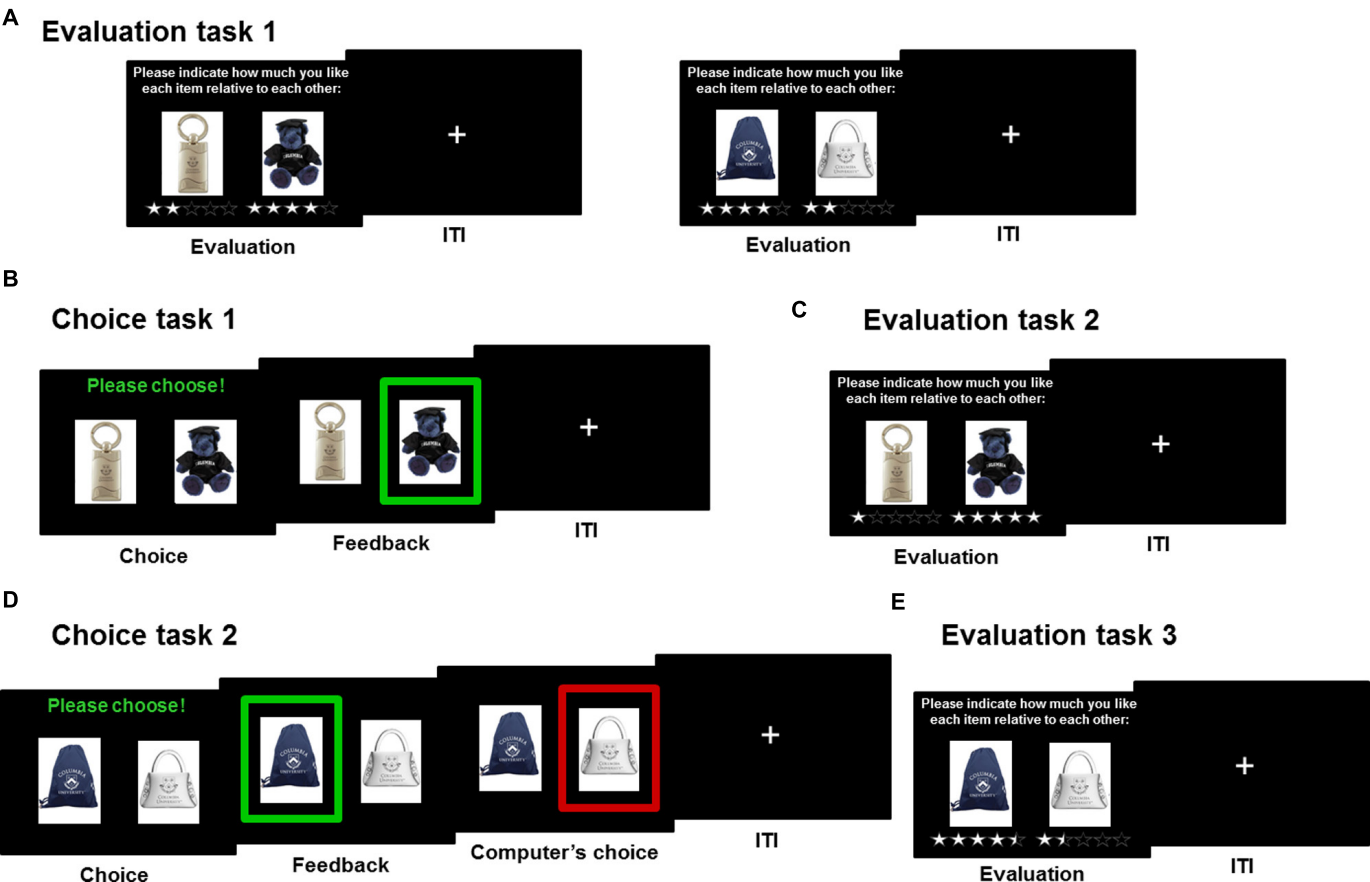
**Experimental design illustration.** **(A)** Participants first evaluated objects relative to one another. **(B)** In Choice task 1, they were asked to choose the object they liked most. **(C)** Subsequently, they evaluated again the pairs of objects presented during Choice task 1. **(D)** In Choice task 2, participants made choices again between two objects but one of the two objects was then selected by the computer. **(E)** Finally, participants evaluated the pairs of objects presented during Choice task 2.

Drawing on the associative self-anchoring account and psychological reactance theory, we hypothesized that people with a high sense of explicit self-esteem would exhibit enhanced evaluations for chosen objects that they might obtain compared to chosen objects that are not obtainable, whereas people with low explicit self-esteem would exhibit enhanced evaluations for chosen objects that are not obtainable compared to chosen objects that are obtainable. To test these predictions, we designed a paradigm where participants were asked to indicate relative evaluations for one of two objects presented side-by-side before and after choosing one of them in conditions where chosen objects were obtainable (i.e., one of the chosen object would randomly be selected by the computer and given to them at the end of the experiment) or not (**Figure 1**).

## Materials and Methods

### Participants

Twenty-eight Columbia University students (15 females) with a mean age of 22.71 ± 4.40 participated in the study. All participants were free of neurological or psychiatric disorders and had normal or correct-to-normal vision. Written informed consent was obtained from all participants, and the study was approved by the Columbia University research ethics committee.

### Experimental Procedure

The experiment was performed simultaneously by two gender-matched participants in two separate testing rooms. Upon arrival, participants were told that they would be administered the same task at the same time. The experiment included five different tasks and lasted approximately 1 h, followed by a short post-experiment questionnaire and the single item self-esteem questionnaire (Robins et al., 2001). In this questionnaire, participants had to indicate by moving a cursor along a 1–7 Likert scale (increments of 1) to what extent the statement “I have high self-esteem” was true of them (mean score = 5.28 ± 1.06).

Stimuli consisted of 280 objects from the Columbia University bookstore, which were presented in pairs, two objects at a time side-by-side (**Figure 1**).

#### Evaluation Task 1

To assess participants’ relative evaluations of all 140 pairs of objects, we asked them to imagine how much they would prefer to obtain each object relative to the other object at the end of the experiment (**Figure 1A**). These evaluations were relative in that increasing the evaluation of one object (i.e., by adding more stars) automatically decreased the evaluation of the other object (i.e., by removing stars). Participants were instructed about the meaning of each star combination, namely: three stars for each object indicated an equal preference, 2.5 stars for one of the objects versus 3.5 stars for the other object indicated a moderate preference, 2 stars versus 4 stars indicated a strong preference for one of the objects over the other object, 1.5 stars versus 4.5 stars indicated a very strong preference and 1 star versus 5 stars indicated an extremely strong preference. Evaluations were made in a self-paced manner, separated by an inter-trial interval (ITI) of 1–5 s. All pairs of items were assigned randomly across subjects. Each item was only presented once.

The purpose for Evaluation task 1 was twofold. First, it provided us with relative evaluations for each pair of objects, and second, it allowed us to extract only those pairs of objects for which a change in evaluation could be optimally detected. Indeed, pairs of objects for which one of the two objects was very strongly to extremely strongly preferred (i.e., 1.5 versus 4.5 stars, or 1 versus 5 stars) were not optimized to allow for an evaluation enhancement of the favored object because of a ceiling effect, while pairs of objects with equal evaluations (i.e., three stars for each object) were not optimal to trigger reactant behavior, which is expressed by an increase in the attractiveness of the unobtainable alternative proportional to its original relative evaluation (Brehm, 1966). Thus, 80 pairs of objects for which one of the two objects was moderately to strongly preferred (i.e., 2.5 versus 3.5 stars, or 2 versus 4 stars) were selected and included in subsequent tasks. If 80 pairs of objects with these combinations were not available for any given participant, we selected the number of pairs with these combinations that were available, using a criterion of a minimum of 50 pairs (note that all participants met this criterion). On average, participants were presented with 64.61 ± 13.64 choice pairs (median = 64).

The rationale for having participants make initial preference ratings between two items presented at a time rather than have them rate each item independently as is usually done in cognitive dissonance experiments (Sharot et al., 2009, 2012; Izuma et al., 2010; Jarcho et al., 2011; Qin et al., 2011; Kitayama et al., 2013) was to avoid the possible confounds raised by Chen and Risen (Chen and Risen, 2010; Izuma and Murayama, 2013). Indeed, in the conventional “Free Choice Paradigm,” participants are first asked to provide individual ratings for a number of items. They are then asked to make choices between two items that have either similar ratings (“difficult choices”) or significantly different ratings (“easy choices”), before re-evaluating each of these items separately. A critical issue associated with this paradigm is that the two items previously rated independently are then evaluated side-by-side at the time of choice, which provides additional information about true preference, and bias measured attitude change following choices. Accordingly, it was shown that the spread of alternative observed following difficult choices could occur in the absence of a preference change and thus, without experiencing dissonance (Chen and Risen, 2010; Izuma and Murayama, 2013). Note that Izuma and Murayama (2013) demonstrated that choices involving differently valued alternatives were not affected by spurious dissonance effects in this paradigm. Thus, in the current experiment, possible confounds were avoided by (1) measuring true preference by having participants evaluate the items relative to one another and (2) making choices between differently valued alternatives.

#### Choice Task 1

Participants were presented with 40 of the 80 pairs of objects selected in Evaluation task 1, presented in a random order. On each trial, they were asked to make spontaneous decisions and were given 3 s to choose the object they would most prefer obtaining at the end of the experiment. Their selection was then highlighted by a green frame for 2 s and the trial ended with a 1–5 s jittered ITI (**Figure 1B**). Importantly, participants were instructed that at the end of the experiment, one of the selected objects throughout the experiment would be randomly picked by the computer and given to them.

#### Evaluation Task 2

Participants were then asked to re-evaluate these 40 pairs of items, like they did in Evaluation task 1 (**Figure 1C**). All pairs of objects were presented in a random order.

#### Choice Task 2

The procedure was identical to Choice task 1, the only exception being that after making their choice, a red frame appeared on the screen for 2 s (**Figure 1D**). Participants were told that this task was synchronized with the other participant and that on each trial the computer would randomly select either their own choice, or that of the other participant. Importantly, participants were instructed to pay close attention to the objects highlighted in red because the object they would receive at the end of the experiment would be randomly selected among the objects they selected during Choice task 1 or amongst the items highlighted in red during Choice task 2. In reality, the tasks were not synchronized and the choices highlighted in red were pre-determined so that half the time the feedback would be congruent with the participant’s choice, and half the time, it would be incongruent with their choice.

This task consisted of 80 trials, including the 40 pairs of objects used in Choice task 1 and the remaining 40 pairs of objects selected after Evaluation task 1. They were all presented in a random order. Note that the change in evaluation did not differ between the pairs of objects used in both Choice tasks versus the pairs of objects used in Choice task 2 only [paired *t*-test, *t*(27) = 0.22, *p* = 0.89], thus further analyses do not distinguish between these pairs of objects.

Note that the order of the sessions and the frame color highlighting the participant’s selection were not counterbalanced across subjects and choice tasks, which might be potential limitations to the current design.

#### Evaluation Task 3

Participants were asked to re-evaluate the pairs of objects used in Choice task 2, like they did in previous Evaluation tasks (**Figure 1E**). All pairs of objects were presented in a random order.

##### Quantifying evaluation change

For each pair of objects presented, the evaluation of one object was made relative to the other object (i.e., an increase in the evaluation of one object necessarily implied a decrease in the evaluation of the other object). Therefore, we only report the evaluation change for the chosen object.

#### Evaluation Change Elicited by Choice Task 1

For each trial *t*, Evaluation Change Scores for Choice task 1 (ECS-1) were computed as follows:

ECS-1(*t*) = rating from Evaluation task 2 for chosen object (*t*) – rating from Evaluation task 1 for chosen object (*t*).

To create an ECS-1 for each participant *P*, we averaged ECS-1 across all trials for that particular participant as follows:

ECS-1-M(*P*) = mean (ECS-1(*t*)).

#### Evaluation Change Elicited by Choice Task 2

Based on participants’ reports from previous pilot studies, ‘congruent’ trials (i.e., when the computer selected the chosen object) were excluded from analyses reported in the Results section. Despite being obtainable, chosen objects in congruent trials elicited various interpretations across participants. Some participants thought that it meant that the computer had randomly elected their own choice every time while in reality it could also mean that the computer had selected the other participant’s choice (which was similar to theirs). In addition, and regardless of this distinction, some participants reported being happy when the computer’s choice was congruent with theirs because that meant that they still had a chance to obtain the chosen object. On the other hand, some participants reported being unsatisfied during Choice task 2 in general because they had no control over the outcome. Therefore, given the multitude of interpretations possible for congruent trials and the different cognitive processes that might have been involved during these trials, they were not considered further. Note that in the case of incongruent trials, participants interpreted the fact that their choice was not selected as the computer having selected the other participant’s choice, as explained in the instructions. Thus, incongruent trials did not involve analogous possible misinterpretations.

Evaluation Change Scores for Choice task 2 (ECS-2) were computed as follows for each trial *t*:

ECS-2(*t*) = rating from Evaluation task 3 for chosen object (*t*) – rating from Evaluation task 1 for chosen object (*t*).

Note that computing ECS-2(*t*) using the rating from Evaluation task 2 for the chosen object (*t*) when available (that is for the choice pairs used in Choice Task 1) did not qualitatively alter our results (2x2 ANOVA, factor Evaluation change: [*F*(1,55) = 0.01, *p* = 0.91], factor explicit self-esteem: [*F*(1,55) = 0.43, *p* = 0.51], interaction: [*F*(1,55) = 5.47, *p* = 0.02].

To create an ECS-2 for each participant *P*, we averaged ECS-2 across all trials for that particular participant as follows:

ECS-2-M(*P*) = mean (ECS-2(*t*)).

For completeness, we conducted similar analyses to the one reported in the Results section replacing ECS-1-M with either (1) the congruent trials from Choice task 2 alone, or replacing it with (2) ECS-1-M + congruent trials from Choice task 2. The results from these 2x2 ANOVAs did not qualitatively differ from the analysis reported in Section “Results” and the interaction between the factors Evaluation change and explicit self-esteem remained virtually significant in both analyses Analysis 1: no main effect for the factor Evaluation change [*F*(1,55) = 0.74, *p* = 0.39] or the factor explicit self-esteem [*F*(1,55) = 2.9, *p* = 0.09], interaction between these two factors [*F*(1,55) = 3.55, *p* = 0.06]; Analysis 2: no main effect for the factor Evaluation change [*F*(1,55) = 0.36, *p* = 0.55] or the factor explicit self-esteem [*F*(1,55) = 0, *p* = 0.97], interaction between these two factors [*F*(1,55) = 3, *p* = 0.08].

### Statistical Analysis

All statistical tests were performed using Matlab 7.9.0 (R2009b). The linear regression modeling the relationship between the evaluation of chosen objects (dependent variable) and explicit self-esteem (independent variable) was performed using the *corrcoef* function. The 2x2 ANOVA with the factors Evaluation change (ECS-1-M versus ECS-2-M) and explicit self-esteem (High versus Low self-esteem) was performed using the *anova2* function. One-sample *t*-tests were performed using the *ttest* function, and two-sample *t*-tests were performed using the *ttest2* function. Note that all statistical tests are two-tailed unless stated otherwise.

## Results

**FIGURE 2:**
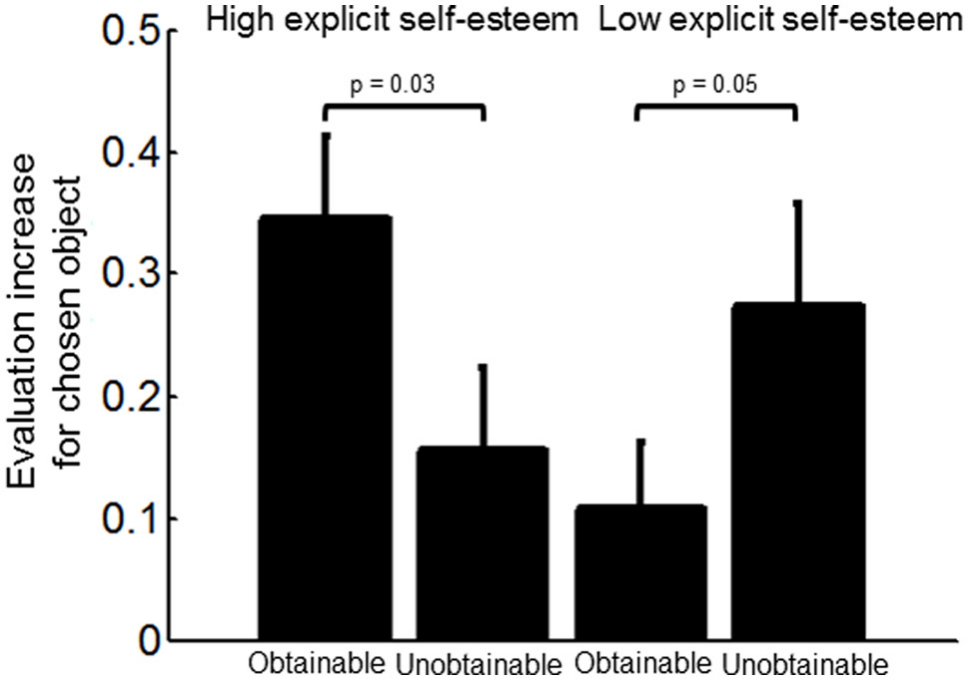
**Interaction between the factors evaluation change of chosen objects according to their obtainibility (ECS-1-M versus ECS-2-M) and explicit self-esteem (Low versus High group; 2x2 ANOVA).** Participants in the high explicit self-esteem group showed more evaluation change of chosen objects when they were obtainable [one-tailed paired *t*-test, *t*(26) = 2.02, *p* = 0.03] while those in the low explicit self-esteem group showed increased preference for chosen objects that were unobtainable [one-tailed paired *t*-test, *t*(26) = -1.67, *p* = 0.05]. Error bars represent SEM.

As predicted, the evaluation of chosen objects that were obtainable (ECS-1-M) was enhanced as a function of explicit self-esteem (linear regression; *r* = 0.45, *p* < 0.05). Additionally, we conducted a 2x2 ANOVA with the factors Evaluation change (ECS-1-M versus ECS-2-M) and explicit self-esteem (High versus Low self-esteem) to test for differences between participants with high versus low explicit self-esteem in their tendency to enhance their evaluation of chosen objects depending on their obtainability. A median split was used to create the High self-esteem group (14 participants) and the Low self-esteem group (14 participants). This analysis revealed no main effect for the factor Evaluation change [*F*(1,55) = 0.03, *p* = 0.87] or the factor explicit self-esteem [*F*(1,55) = 0.77, *p* = 0.38; **Figure 2**]. As expected, we found a significant interaction between these two factors [*F*(1,55) = 6.75, *p* = 0.01, $\eta_{p}^{2}$ = 0.11], such that participants in the high self-esteem group exhibited more Evaluation change for chosen objects that were obtainable compared to chosen objects that were unobtainable [*M* = 0.19, *SD* = 0.33, *t*(13) = 2.13, *p* = 0.05, *d* = 0.77] whereas participants in the low self-esteem group exhibited more evaluation change for chosen objects that were unobtainable compared to those that were obtainable [*M* = 0.17, *SD* = 0.23, *t*(13) = 2.67, *p* = 0.02, *d* = 0.59].

Interestingly, when asked in a post-experiment questionnaire whether they felt that their evaluations had changed following either Choice task, participants reported that their relative evaluation for the chosen object remained unchanged following Choice task 1 [*M* = 5.46, *SD* = 1.73, *t*(27) = 1.42, *p* = 0.17, one sample *t*-test comparing the mean to the neutral value 5 on our 1–9 Likert scale] and Choice task 2 [*M* = 5, *SD* = 1.72, *t*(27) = 0, *p* = 1, one sample *t*-test comparing the mean to the neutral value 5 on our 1–9 Likert scale]. This suggests that evaluation change occurred without participants’ awareness, and regardless of the obtainability of the chosen object.

## Discussion

The present results provide preliminary evidence indicating that obtainability of chosen objects differentially impacts the evaluation of these objects according to one’s sense of explicit self-esteem. Specifically, participants with high self-esteem increased their relative evaluation of chosen objects further when they may be obtained in the future as compared to when they were unobtainable, whereas participants with low self-esteem showed the reverse pattern.

First, we provide evidence suggesting that explicit self-esteem modulates evaluations of chosen objects that are obtainable in a self-serving way, extending prior findings demonstrating a similar modulation by implicit self-esteem (Gawronski et al., 2007). This finding accords well with the idea that the act of choosing an object that we may later belong to the self generates an association between this object and the self, giving rise to a transfer of self-evaluation to the chosen object, a phenomenon referred to as associative self-anchoring (Greenwald and Banaji, 1995; Cadinu and Rothbart, 1996; Walther and Trasselli, 2003; Gawronski et al., 2007). Consistent with the idea that associative self-anchoring is independent from dissonance reduction processes (Gawronski and Bodenhausen, 2006; Gawronski et al., 2007), all objects within each pair of objects used in the current study were differently valued and thus, should not elicit cognitive dissonance (Festinger, 1957; Harmon-Jones and Mills, 1999). Indeed, it has long been argued that having to choose between equally attractive alternatives generates cognitive dissonance which will be reduced through a spreading-of-alternatives effect, whereby positive features of the chosen alternative and negative features of the rejected alternative are emphasized (Brehm, 1956; Frey, 1986; Olson and Stone, 2005), an effect that has not received support in the case of differently valued alternatives (Brehm, 1956; Sharot et al., 2009; Kitayama et al., 2013).

Furthermore, our findings might relate to psychological reactance theory which, applied to the current case, would predict that eliminating the possibility of receiving a chosen object increases the attractiveness of this object (Brehm, 1966; Brehm and Brehm, 1981). Consistent with previous findings showing a negative relationship between explicit self-esteem and reactant behavior (Joubert, 1990), we found that participants with low explicit self-esteem exhibited enhanced evaluations for chosen objects that were unobtainable compared to objects that were obtainable, an effect that was reversed in participants with high explicit self-esteem. Note that in the case of threat to freedom (as opposed to elimination of freedom as is the case in the current study), self-esteem has been shown to correlate positively with reactant behavior (Brockner and Elkind, 1985); however it is not surprising that the relationship between self-esteem and psychological reactance reverses depending on whether there is a possibility for freedom restoration.

One limitation of the current research is the relatively small sample size (*n* = 28) which leads our results to be preliminary. The fact that explicit self-esteem was measured at the end, rather than at the beginning of the experiment, places another potential limitation. Indeed, performing the task might have somehow modulated participants’ sense of self-esteem, resulting in a measure of state explicit self-esteem rather than trait explicit self-esteem. However, given that the questionnaire explicitly examines trait self-esteem, and that the paradigm should not lower self-esteem at least to the capacity where it would drive the robust results presented here, we expect that any state effect to be negligible. Also, we cannot rule out the possibility that some of the choices might have been surprising to the participant. However, given that (1) choices are subjective in nature, (2) the two participants performing the experiment simultaneously did not know each other, and (3) the items we used in the experiment were all within a narrow price range, we believe that suspicions were kept to a minimum and did not interfere with the purpose of the experiment. Finally, it is important to note that the social impact of decisions (i.e., other’s rejection of an item) may impact one’s preferences and future studies should probe the effects of social or any other bias.

Linking our findings to positive illusions, participants with high self-esteem distorted the perception of chosen objects in a positive light compared to participants with low self-esteem, such that the relative attractiveness of these objects was enhanced further when there was a possibility for these objects to belong to them (Taylor and Brown, 1988). It is interesting to speculate that the rationalization deficiency exhibited by people with low self-esteem together with their tendency to ruminate over negative consequences might relate to the well-known correspondence between low self-esteem, depression and anxiety (Sowislo and Orth, 2013).

However, it is important to note that in accordance with the consistency bias where past attitudes are incorrectly remembered to resemble present’s attitudes, participants’ reports suggest that they were not aware of the fact that their evaluations changed between the different evaluation tasks. Consistent with the fact that participants were not aware of altering their evaluations, preference change has been shown to be an implicit process (Sharot et al., 2010; Jarcho et al., 2011; Coppin et al., 2012, 2014), an idea further exemplified by studies conducted in monkeys, children and amnesic patients (Lieberman et al., 2001; Egan et al., 2007, 2010). Although some of the dissonance studies cited above suffered from the artifact raised by Chen and Risen (2010) and should be interpreted with caution, there is emerging evidence that the change in evaluation we observe might reflect a non-conscious mechanism serving important adaptive purposes. Indeed, being contented with what might be part of one’s environment, rather than dwelling upon what could have been might act as a positive reinforcement loop, whereby associative self-anchoring induces a positive feeling that further enhances self-esteem, and ultimately well-being (Taylor and Brown, 1988).

## Conflict of Interest Statement

The authors declare that the research was conducted in the absence of any commercial or financial relationships that could be construed as a potential conflict of interest.
